# A novel prognostic model predicting the long-term cancer-specific survival for patients with hypopharyngeal squamous cell carcinoma

**DOI:** 10.1186/s12885-020-07599-2

**Published:** 2020-11-11

**Authors:** Xin Tang, Tong Pang, Wei-feng Yan, Wen-lei Qian, You-ling Gong, Zhi-gang Yang

**Affiliations:** 1 Department of Radiology, West China Hospital, Sichuan University, 37# Guo Xue Xiang, Chengdu, 610041 Sichuan China; 2 Department of Thoracic Oncology and State Key Laboratory of Biotherapy, Cancer Center, West China Hospital, Sichuan University, 37# Guo Xue Xiang, Chengdu, 610041 Sichuan China

**Keywords:** Head and neck cancer, Hypopharyngeal squamous cell carcinoma, Cancer-specific survival, Nomogram, Prognostic model

## Abstract

**Background:**

Hypopharyngeal squamous cell carcinoma (HSCC) is a rare type of head and neck cancer with poor prognosis. However, till now, there is still no model predicting the survival outcomes for HSCC patients. We aim to develop a novel nomogram predicting the long-term cancer-specific survival (CSS) for patients with HSCC and establish a prognostic classification system.

**Methods:**

Data of 2021 eligible HSCC patients were retrieved from the Surveillance, Epidemiology and End Results database between 2010 and 2015. We randomly split the whole cases (ratio: 7:3) into the training and the validation cohort. Cox regression as well as the Least absolute shrinkage and selection operator (LASSO) COX were used to select significant predictors of CSS. Based on the beta-value of these predictors, a novel nomogram was built. The concordance index (C-index), the calibration curve and the decision curve analysis (DCA) were utilized for the model validation and evaluation using the validation cohort.

**Results:**

In total, cancer-specific death occurred in 974/2021 (48.2%) patients. LASSO COX indicated that age, race, T stage, N stage, M stage, surgery, radiotherapy and chemotherapy are significant prognosticators of CSS. A prognostic model based on these factors was constructed and visually presented as nomogram. The C-index of the model was 0.764, indicating great predictive accuracy. Additionally, DCA and calibration curves also demonstrated that the nomogram had good clinical effect and satisfactory consistency between the predictive CSS and actual observation. Furthermore, we developed a prognostic classification system that divides HSCC patients into three groups with different prognosis. The median CSS for HSCC patients in the favorable, intermediate and poor prognosis group was not reached, 39.0-Mo and 10.0-Mo, respectively (*p* < 0.001).

**Conclusions:**

In this study, we constructed the first nomogram as well as a relevant prognostic classification system that predicts CSS for HSCC patients. We believe these tools would be helpful for clinical practice in patients’ consultation and risk group stratification.

## Background

Hypopharyngeal carcinoma is a rare type of cancer accounting for approximately only 2–6% of head and neck neoplasms [1, 2]. However, patients with hypopharyngeal carcinoma harbored very poor clinical prognosis with an estimated 5-year overall survival (OS) rate of around 30–35% [3, 4]. Consistent with other tumors in head and neck, the most common pathological type of hypopharyngeal carcinoma is hypopharyngeal squamous cell carcinoma (HSCC) [5].

Due to its low incidence, only very few studies had investigated the prognostic factors of HSCC. By now, tumor-node-metastasis (TNM) staging system is still the most commonly used method for prognostic evaluation for HSCC patients. Other reported adverse indicators of HSCC included age, performance status, advanced clinical stage [6–9]. However, a major flaw of the existing researches is that, they only focused on the effects of various prognostic factors on the survival time of HSCC patients in isolation, whereas none of them analyzed the patients’ prognosis by comprehensively combining various prognostic factors together.

It is well acknowledged that, because of the heterogeneity of tumor, the prognosis of patients with cancer is affected by various factors. Thus, to more accurately predict the prognosis of HSCC patients, it is essential to evaluate the survival outcomes of patients by integrating multiple risk factors. A nomogram is an ideal tool to achieve this goal. In general, nomograms are the visualization of prognostic models. One of the biggest advantages of nomograms is that, they can conveniently calculate and present the survival probability of a certain time-point for a certain patient based on risk factors included. Hence, nomograms are useful in the prognostic evaluation and are widely used in the real clinical practice in various cancer types [10].

The purpose of this study is to develop a novel nomogram predicting the long-term cancer-specific survival (CSS) for patients with HSCC. In addition, we also established a prognostic classification system which can divide patients into different prognostic subgroups.

## Methods

### Patients selection and data collection

Patients’ clinical and prognostic information were all retrieved from the Surveillance, Epidemiology and End Results (SEER) database. The SEER database is a population-based cancer database that collects data from 18 registries among 14 states and covers around 28% population across the USA [11]. Cases included in the current study were all diagnosed with HSCC as the primary malignant tumor between 2010 and 2015. Those with incomplete clinical or prognostic characteristics or with hypopharyngeal carcinoma other than squamous cell carcinoma were excluded. Finally, 2021 eligible cases were included in analyses. The following factors were collected from each patient: age, year of diagnosis, sex, race, American Joint Committee on Cancer (AJCC, the 7th edition) stage, TNM stage, grade and treatment experiences.

### Statistical methods

The primary endpoint and the predicted target of this study is CSS which is the interval from the initial diagnosis of HSCC to death caused by it. Overall survival (OS) was the secondary endpoint which was duration from the initial diagnosis to death of any cause.

The specific processes of prognostic model building and nomogram construction were as follow: Firstly, the total cohort was randomly split (splitting ratio: 7:3) into the training and validation cohort. The development of the prognostic model and the corresponding nomogram was carried out using the training cohort, whereas the validation cohort was responsible for the validation of the model. Secondly, Cox regression was conducted to test factors’ value in predicting CSS. Thirdly, the variable selection process was performed by the least absolute shrinkage and selection operator (LASSO) COX. Fourthly, the beta-value of predictors was calculated by the traditional Cox regression. Finally, based on the beta-value of these factors, the CSS-predicting model was visually presented in the way of nomogram.

The validation of the novel nomogram was conducted in three distinct aspects using the validation cohort, i.e. the discrimination power, the calibration and the clinical effect. The discrimination power was assessed by the C-index. Calibration curves were carried out to evaluate the calibration of the model in diverse time-points with 500 bootstrap resamples. In addition, decision curve analyses (DCAs) were utilized to examine if the prognostic model was useful in clinical decision making.

We also developed a CSS prognostic classification system in corresponding to the nomogram. According to the total score of each patient calculated by the nomogram from low to high, all cases could be classified into three groups. Each with roughly the same number of patients and then all cases could be classified into the favorable, intermediate and poor prognostic group.

The chi-square test or the Mann–Whitney rank-sum test was conducted to compare the baseline factors between cases in the training and the validation cohort. Survival outcomes were compared by the log-rank test and were exhibited by Kaplan-Meier curves. Data retrieving was conducted by using SEER*Stat (version 8.3.5). R software (version 3.6.1) and MedCalc statistical software 15 were used for data analyses. All tests were two-sided. A *p* value < 0.05 was considered statistically significant for all the tests.

## Results

### Patients’ characteristics

In total, 2021 patients were included in this study. Finally, cancer-specific death and all-cause death occurred in 974/2021 (48.2%) and 1236/2021 (61.2%) patients, respectively. The median CSS and OS of the total patients were 32.0-Mo (95%CI: 26.9–37.1 Mo) and 23.0-Mo (95%CI: 20.7–25.3 Mo), respectively. The total cases were randomly split into the training cohort (1415, 70%) and validation (606, 30%) cohort as is shown in Table 1. The baseline characteristics between those in the training and validation cohort were perfectly balanced. Besides, the survival outcomes were also matched between the two groups (Median CSS: 31.0- vs. 38.0-Mo, *p* = 0.215; Median OS: 23.0 vs. 22.0-Mo, *p* = 0.951).

**Table 1:** Baseline characteristics of patients in the training and the validation cohort

	All cohort (***N*** = 2021)	Training cohort (***N*** = 1415)	Validation cohort (***N*** = 606)	***P*** value
**Age**				
**Median (IQR)**	63 (56–70)	63 (56–70)	63 (56–71)	0.606
**Year of Diagnosis**				
**2010**	344 (17.0%)	227 (16.0%)	117 (19.3%)	0.450
**2011**	335 (16.6%)	234 (16.5%)	101 (16.7%)	
**2012**	356 (17.6%)	261 (18.4%)	95 (15.7%)	
**2013**	364 (18.0%)	255 (18.0%)	109 (18.0%)	
**2014**	319 (15.8%)	227 (16.0%)	92 (15.2%)	
**2015**	303 (15.0%)	211 (14.9%)	92 (15.2%)	
**Sex**				
**Male**	1681 (83.2%)	1177 (83.2%)	504 (83.2%)	0.995
**Female**	340 (16.8%)	238 (16.8%)	102 (16.8%)	
**Race**				
**White**	1521 (75.3%)	1061 (75.0%)	460 (75.9%)	0.906
**Black**	352 (17.4%)	249 (17.6%)	103 (17.0%)	
**Other**	148 (7.3%)	105 (7.4%)	43 (7.1%)	
**Grade**				
**Grade I-II**	853 (42.2%)	601 (42.5%)	252 (41.6%)	0.909
**Grade III-IV**	687 (34.0%)	477 (33.7%)	210 (34.7%)	
**Unknown**	481 (23.8%)	337 (23.8%)	144 (23.8%)	
**AJCC Stage**				
**I**	71 (3.5%)	44 (3.1%)	27 (4.5%)	0.221
**II**	172 (8.5%)	118 (8.3%)	54 (8.9%)	
**III**	357 (17.7%)	241 (17.0%)	116 (19.1%)	
**IV**	1421 (70.3%)	1012 (71.5%)	409 (67.5%)	
**T stage**				
**T1**	194 (9.6%)	132 (9.3%)	62 (10.2%)	0.722
**T2**	692 (34.2%)	491 (34.7%)	201 (33.2%)	
**T3**	509 (25.2%)	349 (24.7%)	160 (26.4%)	
**T4**	626 (31.0%)	443 (31.3%)	183 (30.2%)	
**N stage**				
**N0**	480 (23.8%)	328 (23.2%)	152 (25.1%)	0.561
**N1**	380 (18.8%)	263 (18.6%)	117 (19.3%)	
**N2**	1036 (51.3%)	731 (51.7%)	305 (50.3%)	
**N3**	125 (6.2%)	93 (6.6%)	32 (5.3%)	
**M stage**				
**M0**	1839 (91.0%)	1280 (90.5%)	559 (92.2%)	0.199
**M1**	182 (9.0%)	135 (9.5%)	47 (7.8%)	
**Treatment**				
**Surgery**				
**Yes**	356 (17.6%)	252 (17.8%)	104 (17.2%)	0.726
**No**	1665 (82.4%)	1163 (82.2%)	502 (82.8%)	
**Radiotherapy**				
**Yes**	1642 (81.2%)	1148 (81.1%)	494 (81.5%)	0.838
**No**	379 (18.8%)	267 (18.9%)	112 (18.5%)	
**Chemotherapy**				
**Yes**	1434 (71.0%)	1015 (71.7%)	419 (69.1%)	0.240
**No**	587 (29.0%)	400 (28.3%)	187 (30.9%)	

IQR: interquartile range

### The prognostic analyses and nomogram construction

**Fig. 1:**
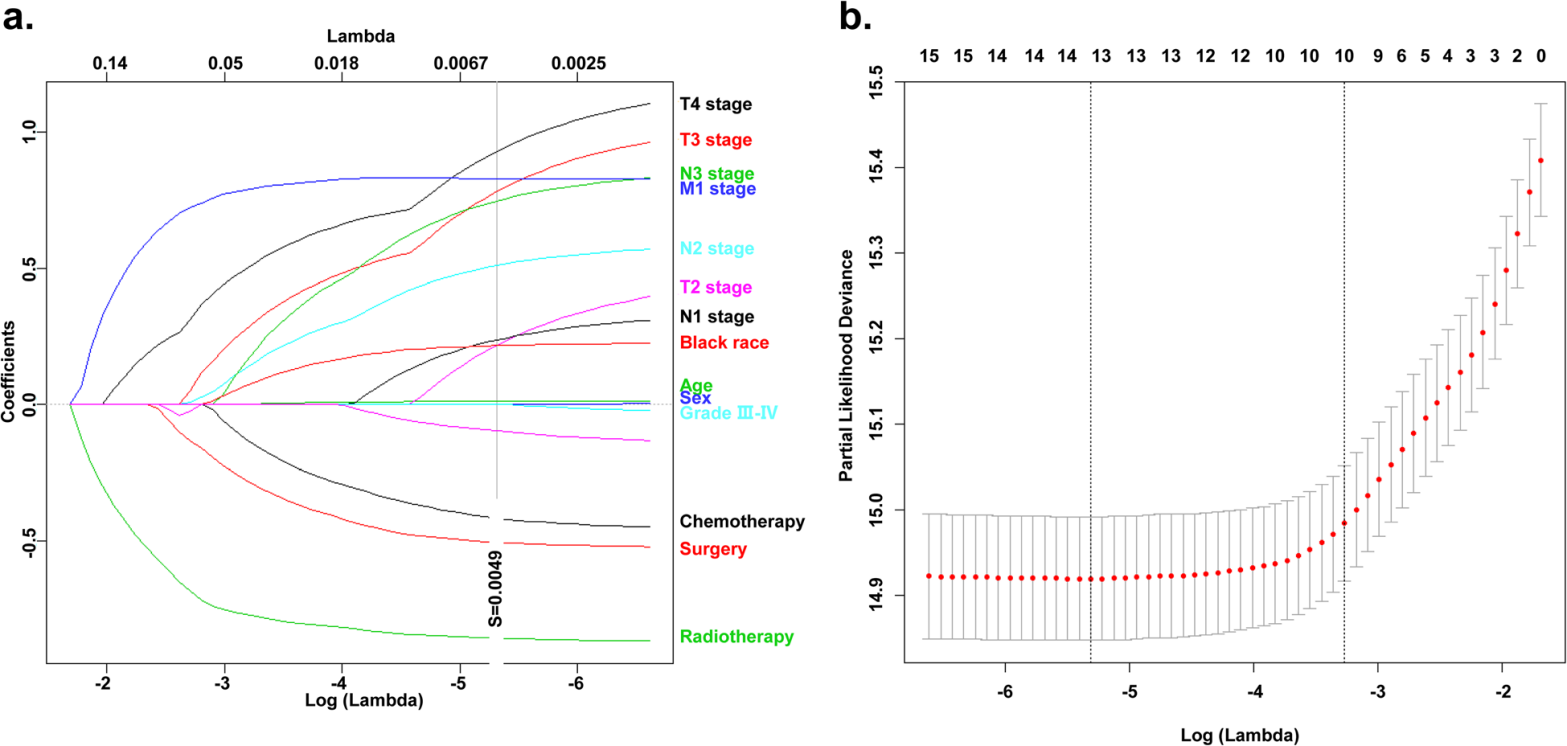
Identifying the prognostic variables of the cancer specific survival (CSS) using the Least absolute shrinkage and selection operator (LASSO) COX. **a** LASSO coefficients of the whole factors included into analysis. The dotted vertical line was drawn at the optimal value choose by the 10-fold cross-validation based on the minimum criteria (the smallest partial likelihood deviance). **b** Tuning parameter identification using the minimum criteria

**Fig. 2:**
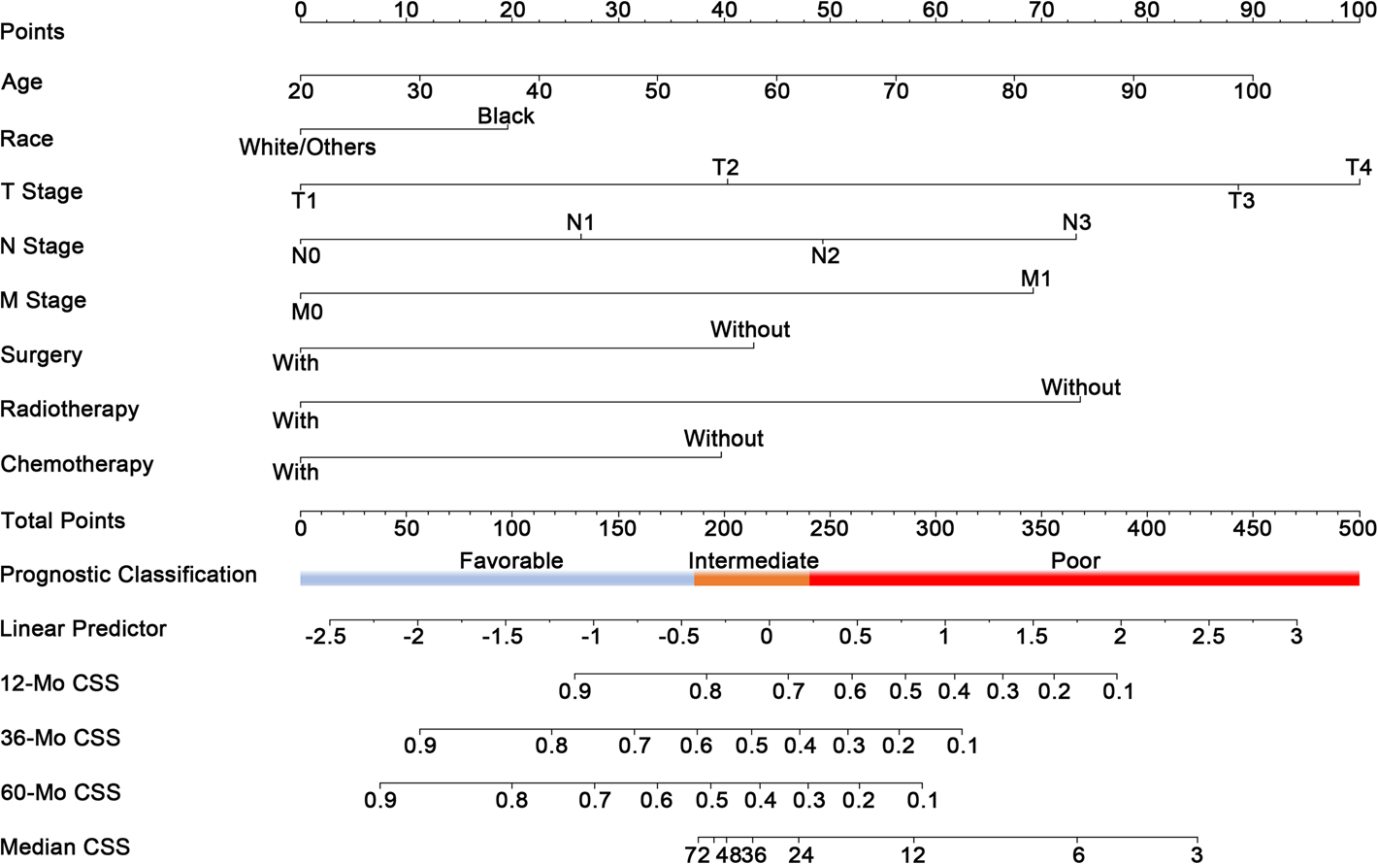
Nomogram that predicts the cancer specific survival (CSS) of HSCC patients. The “total points” of a certain patient is calculated by adding all the scores of the 8 predictors. Based on the total points, the possibility CSS at different timepoints (12-Mo, 36-Mo and 60-Mo) and the prognostic group is obtained. The median CSS time can also be calculated

The prognostic significance of each factor in predicting CSS is evaluated using the training cohort. Statistically significant predictive value was achieved among factors including age, race, AJCC Stage, T stage, N stage, M stage, surgical treatment, radiotherapy treatment and chemotherapy in univariate analyses (Table 2). These factors were also good predictors of OS (Data unshown). Furthermore, variable selection was conducted by the LASSO COX in preparation for the nomogram construction (Fig. 1a, b). In this course, a vertical line indicates the value chosen by 10-fold cross-validation according to the minimum criteria which is the value of λ associated with the lowest partial likelihood deviance. Since the AJCC stage was evaluated based on T, N and M stage, we did not include it into the variable selection to avoid duplicate analysis. Finally, the above-mentioned factors were all included in the prognostic model building. Based on the beta-value of these variables calculated by the multivariate COX regression (Table 3), the prognostic model was visually presented in the way of nomogram (Fig. 2).

**Table 2:** Univariate Cox proportional regression of each factors’ value in predicting CSS

	Bate value	HR	95%CI of HR	***P*** value
**Age (Continuous variable)**	0.010	1.011	1.003–1.018	0.006
**Sex**				
**Female vs. Male**	−0.154	0.858	0.699–1.052	0.141
**Race**				
**White vs. Black**	−0.361	0.697	0.578–0.840	< 0.001
**Other vs. Black**	−0.347	0.706	0.512–0.975	0.034
**Grade**				
**Grade III-IV vs. Grade I-II**	0.000	1.000	0.843–1.188	0.997
**AJCC Stage**				
**II vs. I**	0.928	2.529	0.981–6.519	0.055
**III vs. I**	1.400	4.055	1.648–9.978	0.002
**IV vs. I**	2.009	7.457	3.091–17.988	< 0.001
**T stage**				
**T2 vs. T1**	0.539	1.715	1.195–2.460	0.003
**T3 vs. T1**	1.072	2.922	2.037–4.191	< 0.001
**T4 vs. T1**	1.313	3.717	2.613–5.287	< 0.001
**N stage**				
**N1 vs. N0**	0.176	1.193	0.924–1.540	0.176
**N2 vs. N0**	0.494	1.639	1.341–2.002	< 0.001
**N3 vs. N0**	0.826	2.284	1.666–3.131	< 0.001
**M stage**				
**M1 vs. M0**	1.259	3.523	2.861–4.338	< 0.001
**Surgery**				
**Yes vs. No**	−0.416	0.660	0.533–0.816	< 0.001
**Radiotherapy**				
**Yes vs. No**	−1.044	0.352	0.297–0.418	< 0.001
**Chemotherapy**				
**Yes vs. No**	−0.389	0.678	0.576–0.797	< 0.001

*HR* Hazard ratio, *CI* Confidence interval, *CSS* Cancer-specific survival

**Table 3:** Multivariate Cox proportional regression of each factors’ value in predicting CSS

	Bate value	HR	95%CI of HR	***P*** value
**Age (Continuous variable)**	0.014	1.014	1.006–1.022	0.001
**Race**				
**White and Other vs. Black**	0.236	1.266	1.049–1.528	0.014
**T stage**				
**T2 vs. T1**	0.486	1.625	1.129–2.340	0.009
**T3 vs. T1**	1.067	2.905	2.012–4.197	< 0.001
**T4 vs. T1**	1.204	3.334	2.328–4.776	< 0.001
**N stage**				
**N1 vs. N0**	0.319	1.376	1.060–1.787	0.017
**N2 vs. N0**	0.594	1.811	1.462–2.244	< 0.001
**N3 vs. N0**	0.882	2.416	1.729–3.376	< 0.001
**M stage**				
**M1 vs. M0**	0.834	2.302	1.844–2.873	< 0.001
**Surgery**				
**Yes vs. No**	−0.516	0.597	0.480–0.743	< 0.001
**Radiotherapy**				
**Yes vs. No**	−0.887	0.412	0.339–0.501	< 0.001
**Chemotherapy**				
**Yes vs. No**	−0.479	0.620	0.511–0.752	< 0.001

*HR* Hazard ratio, *CI* Confidence interval, *CSS* Cancer-specific survival

### The validation of the nomogram

**Fig. 3:**
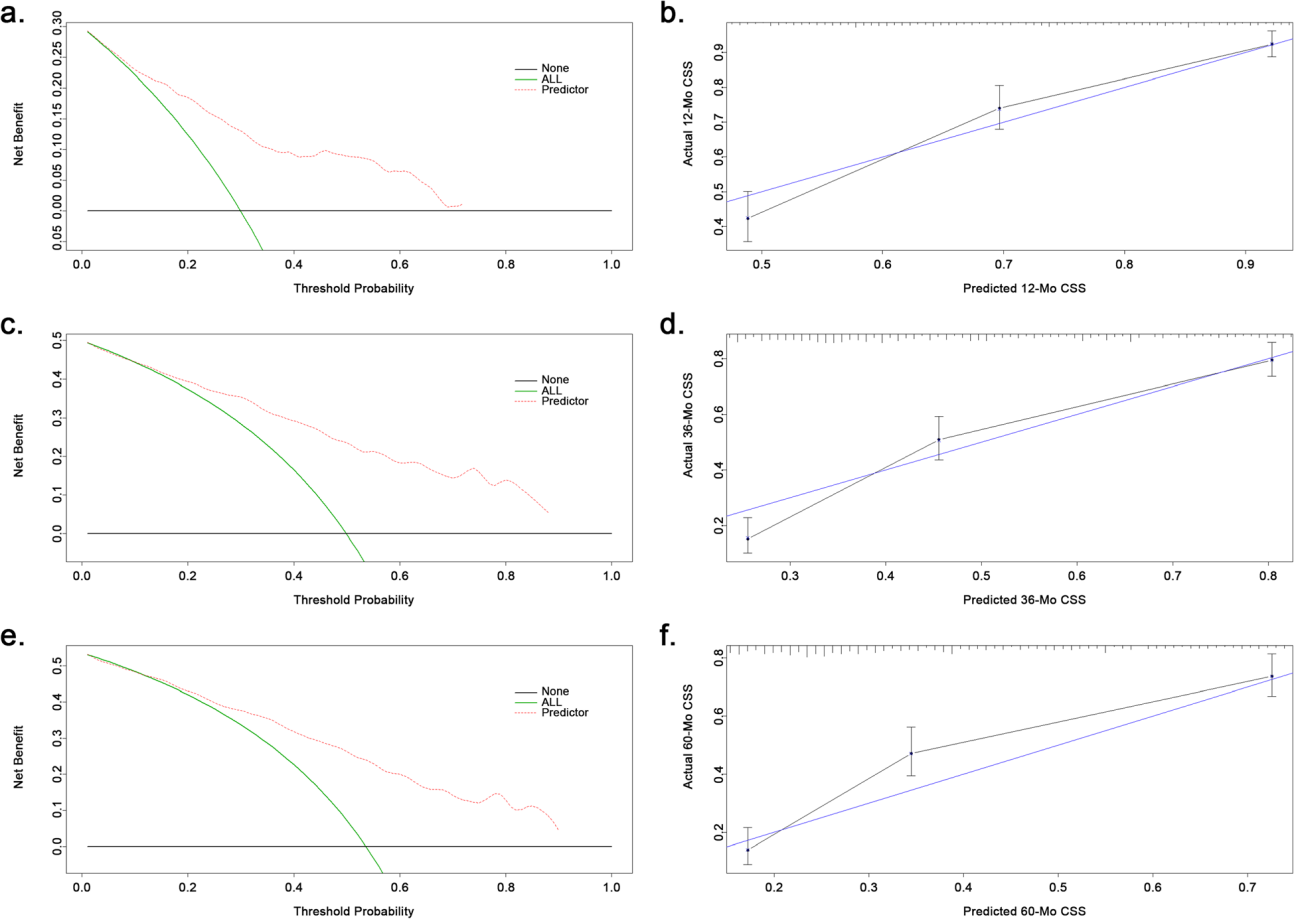
The validation and evaluation of the prognostic model. **a**, **c**, **e** Decision curve analyses of the model predicting cancer specific survival (CSS) at 12-Mo, 36-Mo and 60-Mo. X-axis shows different thresholds. Y-axis represents the net benefit. Net benefit was counted as summing the true positives and subtracting the false positives. The black horizontal line assumes that no patients died whereas the green line assumes all cases dead. **b**, **d**, **f** Calibration curves of CSS at different timepoints (12-, 36- and 60-Mo)

The validation and evaluation of the nomogram was carried out using the validation cohort. The C-index of the prognostic model was 0.764 (95%CI: 0.735–0.793) which was significantly higher than any factor alone or the TNM staging system (C-index: 0.669), indicating that the novel model has a great discrimination ability. Besides, the potential clinical effect of the nomogram was tested using DCA (Fig. 3a, c, e). The results revealed that the model has high positive net benefits among almost all the threshold probabilities at the time-points of 12-Mo, 36-Mo and 60-Mo respectively. Moreover, calibration curves also reflected great consistency between the model prediction and actual observation of the probability of 12-Mo, 36-Mo, and 60-Mo CSS (Fig. 3b, d, f).

### The CSS prognostic classification system

**Fig. 4:**
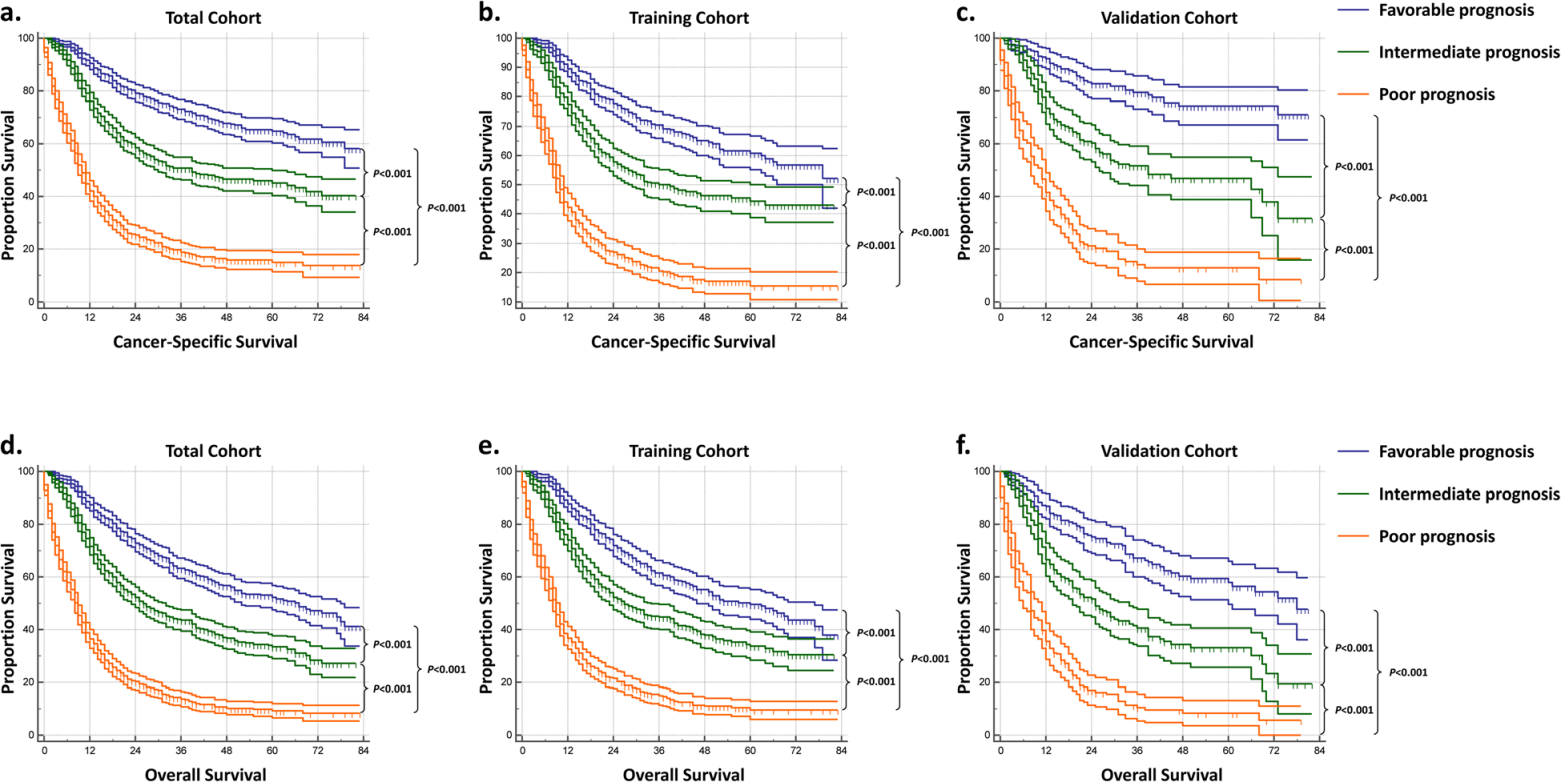
Kaplan–Meier curves showing cancer specific survival (CSS, **a-c**) and overall survival (OS, **d-f**) with their 95% confidence intervals (CIs) of cases in the favorable, intermediate, and poor prognosis group

Apart from the nomogram, we also constructed a corresponding CSS prognostic classification system which could divide all patients into three distinct prognostic groups, i.e. favorable, intermediate and poor prognosis group. During this process, the total score of each patient was calculated by the novel nomogram. According to the total score from low to high, all patients were classified into three groups, each with roughly the same number of patients, i.e. favorable prognosis group (total score: 0–186), intermediate prognosis group (total score: 186–240) and poor prognosis group (total score: 240–500) (Fig. 2). In the whole patients, the median CSS of cases in the favorable, intermediate and poor prognosis group are not reached, 39.0-Mo (95%CI 25.2–52.8 Mo) and 10.0-Mo (95%CI 9.0–11.0 Mo), respectively, whereas the median OS among the three groups are 67.0-Mo (95%CI: 54.5–79.5 Mo), 26.0-Mo (95%CI: 21.8–30.2 Mo) and 9.0-Mo (95%CI: 8.1–9.9 Mo), respectively (*p* < 0.001 between any two groups, Fig. 4a, d). The prognosis among the three prognostic groups were well separated in the training and validation cohort as well (Fig. 4).

## Discussion

HSCC is a distinct tumor type of the head and neck with notoriously poor prognosis. Of note, the survival outcomes varied among patients to patients due to the varied clinical features and therapeutic options. In the current study, based on data of 2021 patients extracted from the SEER database, we built the first model predicting the CSS of HSCC patients. The validation of the novel nomogram demonstrated its good performance in terms of discrimination ability, calibration and clinical usefulness. Additionally, a more simplified prognostic classification system was also generated which could classify all HSCC patients into the favorable, intermediate and poor prognosis group.

Although tumors of the head and neck have great similarities in treatment, their clinical outcomes differed a lot. Among these tumors, HSCC is one of the worst prognostic malignancies. One important reason for this is the unique anatomy of the hypopharynx. Hypopharynx is rich of submucosal lymphatic network, which largely promotes the possibility of tumor metastasis through the lymphatic system [2, 12, 13]. Additionally, the lack of specific early symptoms increases the difficulty of early diagnosis of HSCC [14, 15]. Besides, in most countries the laryngoscope is not a routine medical exam. As a consequence, a great proportion of HSCC patients were confirmed to have an advanced stage disease at the initial diagnosis [16]. It was estimated that, up to 75% of newly diagnosed HSCC patients harbored stage III or IV disease [17], though in this study the proportion is a little bit lower (56.2%). Thus, accurate prognostic evaluation of HSCC patients is essential not only for patients’ consultation, but also, more importantly, for the individualized treatment decision making and then improving patients’ prognosis.

According to a very recent review study summarizing the existing nomograms of head and neck cancers, more than 40 nomograms have been built for tumors including nasopharyngeal carcinoma, oral cavity, oropharynx, larynx salivary gland and etc. [18] There are also nomograms designing for the head and neck tumors as a whole ignoring the specific organ [19–21]. The basis of it is that approximately 90% of patients with head and neck cancers are with squamous carcinoma. However, due to the great heterogeneity among different malignancies, this practice has inevitable defects. Unfortunately, till now, there is no prognostic model or nomogram specifically predicting the survival outcomes of HSCC patients.

In the current study, the first prognostic model predicting the CSS for HSCC patients was built. Our nomogram had a C-index of 0.762, which was higher than most of the other nomograms for head and neck tumors [18]. Furthermore, another advantage of the novel nomogram is that we also built a corresponding prognostic classification system according to the nomogram, which could intuitively provide the actual benefits of a certain treatment to a certain patient. For example, for a 60-year-old black patient with T4N1M0 HSCC, receiving chemoradiotherapy could improve the patient from the poor prognosis group to the intermediate prognosis group. The survival outcomes of cases in different prognostic groups were compared using Kaplan-Meier curves. Interestingly, we found that the survival curves of patents in different prognostic groups were differentiated as well as, or even slightly better, in the validation cohort compared to those in the training cohort. We believe these findings just demonstrated the great discriminating power of the model we developed.

The included variables in the novel nomogram could be divided into two parts, i.e. the clinical factors (age, race, T, N and M stage) and the treatment-related part (whether or not being treated with surgery, radiotherapy and/or chemotherapy). The old age and black race were found to be adversely associated with short CSS in HSCC patients, which was in accordance with other studies [6, 22, 23]. Besides, the great prognostic effect of the TNM staging system was verified again in our study. Remarkably, the predictive accuracy of the novel nomogram was much superior to the TNM staging system (C-index: 0.764 vs. 0.669), indicating that TNM staging alone is not enough for prognostic evaluation.

The nomogram also revealed that treatment is essential to prolong the survival time of HSCC patients. Total laryngectomy used to be the standard way of treatment for patients with HSCC. However, with the development of new therapeutic agents, there was a shift from surgery to the laryngeal preservation therapy after the introduction of chemoradiotherapy [24, 25]. For selected patients, chemotherapy followed by radiation shared similar therapeutic outcomes and regional control to surgical treatment while allow more than half of the HSCC patients to retain the larynx [24]. In our study, less than 20% HSCC patients were treated with surgery, while around 80 and 70% cases chose to receive radiotherapy and chemotherapy, respectively.

It is well acknowledged that the pathogenesis of head and neck cancers is associated with virus infection. For example, Epstein-Barr virus (EBV) and human papilloma virus (HPV) has been identified to be related with the occurrence of oropharyngeal cancer and nasopharyngeal carcinoma, respectively [26, 27]. However, there is no relevant evidence that virus infection correlates with the incidence of HSCC. In addition, virus infection has also been reported as a prognostic factor for survival outcomes for head and neck cancers. For instance, HPV-positive oropharyngeal cancer patients have a longer OS compared with HPV-negative ones, whereas high plasma EBV DNA level was associated with decreased OS for nasopharyngeal carcinoma patients [28–30]. Yet, no evidence implied that virus infection had any effect on the prognosis of HSCC patients. Besides, the SEER database is absence of virus infection data, so we did not analyze the prognostic value of virus infection in our study.

Our study still owns many limitations. First of all, this is a retrospective study with weakness related to its study type. Secondly, although the novel nomogram was generated based on a quite big sample size and a split validation of the model was performed, no external validation using data from other centers was carried out. However, due to the low incidence of HSCC, designing an external validation study is faced with great difficulties. Thirdly, since SEER database does not provide detailed chemotherapy regimens and radiotherapy doses, the prognostic value of these factors could not be assessed. Finally, since all patients included in this study were diagnosed with HSCC between 2010 and 2015, only cTNM stage based on the 7th edition of the AJCC staging system was available in the SEER database, because the AJCC 8th edition was released in late 2017. Therefore, the 7th instead of the latest 8th edition of the AJCC staging system was used for cTNM staging in the current study. However, there are only very little changes in the 8th edition of AJCC clinical staging criteria against the 7th, i.e. in the 8th edition extranodal extension was included in the N stage evaluation, while the T and M stage criteria remained unchanged.

## Conclusion

We constructed the first nomogram that predicts the CSS for HSCC patients in the current study. The validation of the prognostic model using a split cohort showed that the nomogram had high discrimination ability, great calibration and satisfactory clinical effect. We also built a prognostic classification system corresponding to the nomogram which could conveniently classify all HSCC patients into three prognostic groups. We believe these tools would be useful in the real clinical practice for patients’ consultation and risk group stratification.

## Data Availability

The data analyzed in this study are openly available and can be found here: http://www.seer.cancer.gov. The raw data were freely accessible by using the SEER*Stat software (version 8.3.5).

## Abbreviations

OS: Overall survival
HSCC: Hypopharyngeal squamous cell carcinoma
TNM: Tumor-node-metastasis
CSS: Cancer-specific survival
SEER: Surveillance, Epidemiology and End Results
AJCC: American Joint Committee on Cancer
LASSO: Least absolute shrinkage and selection operator
DCAs: Decision curve analyses
HR: Hazard ratio
CI: Confidence interval
HPV: Human papilloma virus
EBV: Epstein-Barr virus
